# Feasibility of alcohol screening among patients receiving opioid treatment in primary care

**DOI:** 10.1186/s12875-016-0548-2

**Published:** 2016-11-05

**Authors:** Anne Marie Henihan, Geoff McCombe, Jan Klimas, Davina Swan, Dorothy Leahy, Rolande Anderson, Gerard Bury, Colum P. Dunne, Eamon Keenan, John S. Lambert, David Meagher, Clodagh O’Gorman, Tom P. O’Toole, Jean Saunders, Gillian W. Shorter, Bobby P. Smyth, Eileen Kaner, Walter Cullen

**Affiliations:** 1 Graduate Entry Medical School, Faculty of Education & Health Sciences, University of Limerick, Limerick, Ireland; 2 UCD School of Medicine, University College Dublin , Belfield, Dublin 4, Ireland; 3 British Columbia Centre for Excellence in HIV/AIDS, St. Paul’s Hospital, 608-1081 Burrard Street, Vancouver, BC V6Z 1Y6 Canada; 4 Addictions Department, Institute of Psychiatry, Psychology & Neuroscience, King’s College London, 4 Windsor Walk, Denmark Hill, London, SE5 8BB UK; 5 Addiction Counsellor, Suite 33, The Morrison Chambers, 32, Nassau Street, Dublin 2, Ireland; 6 Addiction Services, Health Services Executive, Dublin, Ireland; 7 Brown-Alpert Medical School, Providence, Rhode Island USA; 8 CSTAR Centre, University of Limerick (UL), Limerick, Ireland; 9 Trinity Centre for Practice and Healthcare Innovation, School of Nursing and Midwifery, Trinity College Dublin, Dublin, Ireland; 10 Department of Public Health and Primary Care, Trinity College Dublin, Dublin, Ireland; 11 Institute of Health and Society, Newcastle University, Newcastle upon Tyne, UK; 12 Department of Veterans’ Affairs, Washington DC, USA

**Keywords:** Alcohol, Primary care, Screening, Agonist treatment, Methadone, General practice, Implementation, Feasibility, Brief intervention, SBIRT

## Abstract

**Background:**

Identifying and treating problem alcohol use among people who also use illicit drugs is a challenge. Primary care is well placed to address this challenge but there are several barriers which may prevent this occurring. The objective of this study was to determine if a complex intervention designed to support screening and brief intervention for problem alcohol use among people receiving opioid agonist treatment is feasible and acceptable to healthcare providers and their patients in a primary care setting.

**Methods:**

A randomised, controlled, pre-and-post design measured feasibility and acceptability of alcohol screening based on recruitment and retention rates among patients and practices. Efficacy was measured by screening and brief intervention rates and the proportion of patients with problem alcohol use.

**Results:**

Of 149 practices that were invited, 19 (12.8 %) agreed to participate. At follow up, 13 (81.3 %) practices with 81 (62.8 %) patients were retained. Alcohol screening rates in the intervention group were higher at follow up than in the control group (53 % versus 26 %) as were brief intervention rates (47 % versus 19 %). Four (18 %) people reduced their problem drinking (measured by AUDIT-C), compared to two (7 %) in the control group.

**Conclusions:**

Alcohol screening among people receiving opioid agonist treatment in primary care seems feasible. A definitive trial is needed. Such a trial would require over sampling and greater support for participating practices to allow for challenges in recruitment of patients and practices.

**Electronic supplementary material:**

The online version of this article (doi:10.1186/s12875-016-0548-2) contains supplementary material, which is available to authorized users.

## Background

Problem alcohol use, defined as a positive AUDIT (Alcohol Use Disorders Identification Test) score [1], is common among patients attending primary care for opioid agonist treatment. Ryder et al. (2009) estimated that 35 % of patients attending primary care for addiction treatment had problem alcohol use [2], and higher rates have been reported in more specialist addiction treatment centres [3]. Among people receiving addiction treatment, problem alcohol use poses additional challenges as alcohol is associated with increased risk of mortality in this vulnerable population [4]. It impacts adversely on many health issues that commonly affect this population, e.g. chronic hepatitis C infection [5], increased risk of fatal opiate overdose [6] and compromised metabolism of methadone [7]. To address these challenges, psychosocial interventions incorporating alcohol screening and brief intervention (SBI) can effectively identify patients with this problem and guide management [8].

Screening, brief intervention, and referral to treatment (SBIRT) for a range of substance use problems is a cost-effective, comprehensive, and integrated system of early intervention and treatment services for individuals who use tobacco, alcohol, or other drugs [9]. A review of brief, multi-contact behavioural counselling interventions among adult patients attending primary care found such interventions reduced the average number of drinks per week by 13–34 %, increased the proportion drinking at moderate or low risk levels by 10–19 %, concluding such interventions were feasible and potentially highly effective components of an overall public health approach to reducing problem alcohol use [10]. The World Health Organisation (WHO) recommends that health professionals provide alcohol SBI for heavy drinkers, however, it is underused with less than 10 % of those who might benefit from SBI, receiving a brief intervention [11].

The integration of complex interventions which can increase the uptake of screening and brief intervention in primary care is a priority to address problem alcohol use among patients attending primary care for opioid agonist treatment. Primary care is an accessible and frequently used health care service. It offers continued patient centred care and an ideal setting for individual, group and community interventions to encourage health promotion and disease prevention. However, the ability to incorporate such interventions into practice may be challenged through workload, lack of time, knowledge or skills [12]. This is especially the case for problem alcohol use among people receiving opioid agonist treatment. Though common in this population [2], implementation rates of SBIRT are low [13, 14], despite there being increased risks from poly-drug use through additive or synergistic effects [15]. Interventions which promote screening and brief intervention in practice are likely to benefit problem alcohol use among this population [13, 16]. A cross sectional study showed screening and brief intervention were inconsistent and associated with practitioner and system factors (such as lack of time, lack of specialist staff and poor service availability), whereas experience / education and integration with other services were among the key enablers [17]. An educational intervention was subsequently developed to enable GPs to deliver brief interventions for problem alcohol use among people receiving opioid agonist treatment and its possible feasibility, acceptability and usefulness in practice was demonstrated in a pilot study [18]. The PINTA (*P*sychosocial *INT*erventions for problem *A*lcohol use) project has therefore further developed a complex intervention (which includes this educational intervention) to enhance screening and brief intervention.

We aimed to determine if a complex intervention designed to support screening and brief intervention for problem alcohol use among people receiving opioid agonist treatment is feasible and acceptable to healthcare providers and their patients in a primary care setting. The specific objectives were: (i) to develop a multi-sided complex intervention (incorporating practice visits, distribution of best practice guidelines and education), (ii) to explore its feasibility and acceptability, and (iii) to inform the subsequent design of a definitive cluster randomised trial by estimating the possible impact of the intervention on practice (i.e. screening, brief intervention and referral to treatment) and outcomes (i.e. the proportion of patients with problem alcohol use).

## Methods

We used a controlled pre-and-post intervention design to establish the feasibility of a complex intervention to promote SBI for problem alcohol use among people receiving opioid agonist treatment, with cluster randomisation at the level of general practice [14]. Participants (GPs, patients) were surveyed on addiction care processes before and after the intervention (3 months).

The intervention consisted of two key components: (i) an academic group intervening at practices (recruitment, education, study procedures); and (ii) participating GPs treating patients (screening, advice, study procedures).

### Participants

Based on the recommendations for good practice in pilot studies [19, 20], it was estimated that 160 patients (attending 16 general practices) would be adequate to examine the actual recruitment and retention rates (i.e. feasibility), and to provide data on acceptability of study processes and outcome measures, which would inform a future definitive trial [21].

General practitioners (*n* = 16) were selected using random stratified sampling, with geographical location (Health Service Executive Mid-West and Dublin Mid-Leinster regions) and the level of methadone provision training forming the strata in the sampling [14]. To prescribe methadone, GPs are subject to clinical audit and must complete special training, while GPs providing methadone treatment for 15 or more patients are subject to more regular audit and advanced training. GPs who prescribe methadone for less than 15 patients are referred to as “level 1 GPs”, and those prescribing for 15 or more as “level 2 GPs” [21].

Individual participants were recruited by GPs over a 16 week period between December 2013 and April 2014. Participating practices were asked to recruit 10 consecutive patients who were aged 18 years or over and receiving addiction treatment / care (e.g. methadone) at the practice. Patients were excluded from the study if they had language difficulties (i.e., unable to speak, read, and write English sufficiently well to complete study questionnaires), were acutely intoxicated, and/or were cognitively impaired (including severe mental health illness) to the extent that they were unable to provide informed consent to participate. Participating practices were found to have an average timeframe of five weeks to complete recruitment.

The study procedures [21] and sampling framework [14] have been reported previously and recruitment process / subsequent engagement with practices are outlined in Table 1.

**Table 1:** Outline of researcher interaction with recruited practices

• Initial invite / practice recruitment:
o 149 GPs received written invitation to participate. o Invitation outlined: (a) study purpose; (b) remuneration for administrative workload generated; (e) GP / practice requirements on participation and content of the intervention. o Sixteen practices were selected to participate in the study
• Practice Visits: Patient recruitment and baseline data collection was completed over a series of four practice visits.
o Visit 1: Researcher visited practices to outline the study, explain patient recruitment, and provided a resource pack outlining study requirements in greater detail. o Visit 2: Researcher facilitated practices to complete a register of patients attending the practice for management of problem drug use. o Visit 3: Researcher conducted a detailed review of clinical records of participating patients during the baseline data collection phase. GPs were also asked about each patient’s problem alcohol use and drug use by the researcher. o Visit 4: Baseline patient interviews conducted in person or by telephone.
• Three months after the complex intervention had been delivered (see summary box 2), follow-up data was collected (i.e. patient interviews, review of clinical records and GP questionnaires) • The ‘control group’ received the complex intervention upon completion of follow-up data collection.

### Intervention

The complex intervention development was informed by the U.K. Medical Research Council (MRC) ‘Framework for design and evaluation of complex interventions to improve health’ [18]. The complex intervention designed to support screening and brief intervention for problem alcohol use among opioid agonist patients used in the study consisted of: practice visits, distribution of best practice guidelines, brief intervention training (see Table 2).

**Table 2:** PINTA complex intervention description

• A multi-sided complex intervention strategy, incorporating practice visits, distribution of best practice guidelines and education (including CME-approved small group sessions); multimedia educational tools (i.e. DVD); MI (motivational interviewing) related training presentation; and demonstration of intervention implementation to attendees.
• Dissemination of a resource pack which included:
(i) Clinical guidelines for the management of problem alcohol use among problem drug users; (ii) ‘A Quick Question’ HSE (Health Service Executive) leaflet promoting reduced alcohol use; (iii) A multimedia educational video demonstrating screening using AUDIT (Alcohol Use Disorders Identification Test) and delivering a BI (brief intervention) using the ‘FRAMES’ model which identifies key elements of brief intervention: feedback, responsibility, advice, menu of strategies, empathy, and self-efficacy; (iv) Resource manual with up-to-date information on support services to help practices in the management of patients with problem alcohol.
• Educational support for participating GPs following workshop. The specific objectives were to: (i) outline the importance of routine, annual screening of all problem drug users; (ii) encourage use of the full AUDIT questionnaire for patients with positive annual screen; (iii) promote delivery of brief intervention to patients in the ‘hazardous’ / ‘harmful’ category, and referral to specialist services for patients in the ‘dependent’ category.

A staggered intervention design was adopted, whereby participating practices were randomised to receive the complex intervention after baseline data collection. The control group received the complex intervention three months later.

### Data collection

Demographic details (GPs and patients) and data on process / outcome measures (i.e. feasibility, acceptability and possible efficacy), were measured at baseline and follow up (three months) by completing study instruments with GPs and patients, and by reviewing clinical records. Health care professionals at participating practices also completed a postal questionnaire on practice/professional details, experience of training and attitudes.

### Measures of feasibility

We measured the feasibility as numbers of recruited and retained GPs and people receiving opioid agonist treatment in primary care.

### Measures of acceptability

GPs' attitudes towards the provision of care for patients with alcohol use disorders were assessed pre / post intervention. They completed the ‘Short Alcohol and Alcohol Problems Perception Questionnaire’ (SAAPPQ). This 10-item, 7-point Likert-type questionnaire measured the attitudes of professionals towards the provision of care for patients with alcohol use disorders. Total scores range from 10 to 70, with lower scores indicative of more negative attitudes. The role security domain within the SAAPPQ includes 2 sub-domains: role adequacy, and role legitimacy (e.g. “I feel I can appropriately advise my patients about drinking and its effects”; “I feel I have the right to ask patients questions about their drinking when necessary”). Therapeutic commitment involves motivation, task specific self-esteem, and work satisfaction [22]. Within the scales of role security and therapeutic commitment (ratings on a 7-point Likert scale ranging from ‘strongly agree’ to ‘strongly disagree’) means were calculated. See Additional file 1 for SAAPPQ scoring key.

GPs rated the importance of each of five barriers to alcohol screening, specifically: lack of training and education, lack of time, lack of specialist staff, poor service availability and attitude of patients. They rated their answers on a Likert scale from one to five (1 = most important and 5 = least important; composite score 5–25).

Usefulness of training, practice visits, materials, guidelines and remuneration was also measured on a five-point Likert scale (1 = very useful and 5 = not at all useful; composite score 5–25).

In addition, qualitative interviews were conducted with both patients and GPs to determine the acceptability of the complex intervention. Semi-structured interviews were conducted with 14 patients and eight General Practitioners (GPs) who had been purposively sampled from practices that had participated in the feasibility study. The interviews were transcribed verbatim and analysed thematically. The qualitative work is reported in more detail separately [23].

### Process / outcome measures

Though not powered to determine effectiveness, the possible impact of the intervention on care process was measured by examining whether patients had received alcohol screening, brief intervention and referral to a specialist alcohol service in the previous three months (i.e. the study duration). Possible impact of the intervention on care outcomes was measured using the ‘AUDIT-Consumption (AUDIT-C)’, a validated and practical three-item screening test for alcohol use disorders or risky drinking which consists of the first three questions of the AUDIT pertaining to consumption [24]. The selection threshold for positive AUDIT-C scores was ≥ 3 for women and ≥ 4 for men [25, 26].

### Human subjects protection

The Irish College of General Practitioners’ (ICGP) Research Ethics Committee approved the study. During recruitment, GPs informed potential participants of study objectives and procedures, provided written information and asked them to provide their written informed consent to participate. The study was conducted in accordance with relevant ethical standards, the Helsinki Declaration of 1975, as revised in 2000.

### Data analysis

Per-protocol analysis was performed with respect to care process and outcome measures. Means, frequencies and percentages were calculated using Statistical Packages for the Social Sciences (SPSS) version 21.0. To account for potential cluster effects in a future definitive trial, an intracluster correlation coefficient (ICC) for care process and outcome measures was calculated [27].

## Results

### Characteristics of participants

**Fig. 1:**
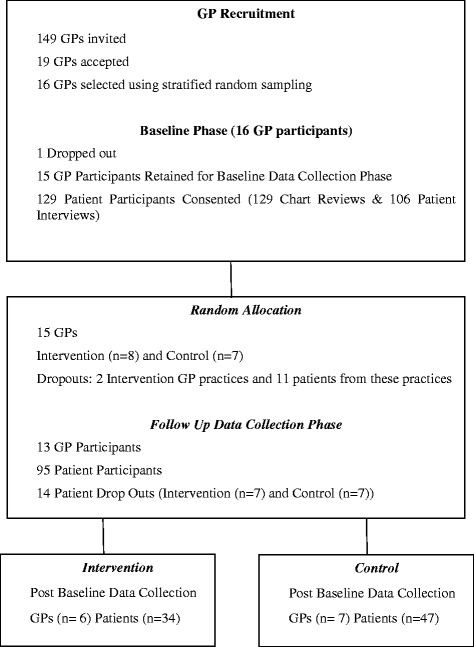
Flow Diagram showing the recruitment of participants from 16 General Practices

Of 16 practices randomly sampled and allocated to either intervention or control groups, 15 facilitated patient recruitment and baseline data collection on 106 patients (Fig. 1). At follow up, 13 practices (Table 3) facilitated data collection on 81 patients (Table 4).

**Table 3:** Participating practices characteristics

Characteristic/Category	GPs Total (*N* = 13)	
	Intervention (*n* = 6)	Control (*n* = 7)
Geographical area of GP:		
Ireland East	5	6
Ireland Mid-West	1	1
Level of training in providing addiction related care:		
Level 1 GP^a^	2	3
Level 2 GP^b^	4	4
Gender of GP:		
Male	5	7
Female	1	0
Type of Practice:		
Mixed^c^	6	6
GMS^d^	0	1
Number of full time GPs per practice:		
≤2	6	5
≥ 3	0	2
Practice Nurse:		
Yes	5	6
No	1	1

^a^Level 1GP: GPs who prescribe methadone for less than 15 patients are referred to as “level 1 GPs”

^b^Level 2 GP: GPs who prescribe methadone for 15 or more are referred to as “level 2 GPs”

^c^Mixed: Mixed Practice accepts both GMS patients and private patients

^d^GMS: General Medical Services Scheme provides free general practitioner services and free drugs and medicines to persons with full eligibility

**Table 4:** Characteristics of patient study population attending intervention and control practices at baseline

Characteristic/Category	Intervention (*n* = 34)		Control (*n* = 47)	
	n (%)	Mean (SD)	n (%)	Mean (SD)
Male	20 (58.8 %)		30 (63.8 %)	
Age		41.5 (7.9)		42.4 (8.8)
Ever injected drugs	23 (67.6 %)		38 (80.9 %)	
Age of first injection		21.3 (3.4)		21.8(6.3)
Hepatitis C positive	20 (58.8 %)		29 (61.7 %)	
Receiving methadone	33 (97.1 %)		47 (100 %)	
Age first methadone use		25.6 (8.3)		25.8 (7.9)
Mean methadone dose		82.2 (22.7)		65.2 (28.6)
Attends Level 1 GP	15 (44.1 %)		26 (55.3 %)	
Attends Level 2 GP	19 (55.9 %)		21 (44.7 %)	
Geographical area of GP:				
East	30 (88.2 %)		43 (91.5 %)	
West	4 (11.8 %)		4 (8.5 %)	
Employed	6 (17.6 %)		2 (4.3 %)	
Current accommodation:				
Rented	9 (26.5 %)		16 (34 %)	
Owned	4 (11.8 %)		4 (8.5 %)	
Family of origin	3 (8.8 %)		8 (17 %)	
Social housing	15 (44.1 %)		17 (36.2 %)	
Supported housing	2 (5.9 %)		2 (4.3 %)	
No Fixed abode	1 (2.9 %)		0 (0 %)	
Past 30-day drug use (self-reported)^a^				
Number of patients who used illicit drugs in the last 30 days^b^	14 (41.2 %)		17 (36.2 %)	
Alcohol screening* *Q: Have you ever been asked about your alcohol use by healthcare professionals?*	19 (55.9 %)		31 (66 %)	
Alcohol brief intervention** *Q: Did they advise you on safe drinking or talk to you about alcohol?*	14 (41.2 %)		23 (48.9 %)	
Specialist referral*** *Q: Have you ever been referred to a specialist/ addiction counsellor for alcohol use?*	7 (20.6 %)		7 (14.9 %)	

^a^Data obtained from baseline patient questionnaires with data referring to the previous 30 days

^b^Heroin, illicit methadone, illicit benzodiazepines, cocaine, amphetamines, cannabis, other drugs

* ICC (SE) = 0.016 (0.014)

** ICC (SE) = –0.06 (0.017)

*** ICC (SE) = 0.22 (0.026)

Overall, the study population (*n* = 81) was similar to those receiving methadone in the national study reporting prevalence of problem alcohol use in Ireland [2]. With the exception of methadone dose (higher in intervention group), both intervention (*n* = 34) and control (*n* = 47) groups were comparable in terms of socio-demographic characteristics and addiction characteristics (Table 4).

### Feasibility

Of 149 invited, 19 GPs expressed an interest and were eligible to participate. Of these 19 practices, we selected 16 by stratified random sampling and 15 of those GPs completed patient recruitment. One hundred and twenty nine patients were recruited, from which we obtained baseline data by reviewing charts (*n* = 129) and interviewing most of the patients (*n* = 106). At follow up, we collected data from 13 GPs. We also collected data by reviewing charts (*n* = 115) and by interviewing 81 of those patients (see Fig. 1).

### Acceptability

Of the seven GPs assigned to the intervention arm, six completed the training and four took part in the educational outreach / practice visits.

The SAAPPQ score increased among both groups between baseline and follow up, but more so in the intervention group (Table 5, +3.3 versus +1.6). Five out of the six GPs in the intervention group revealed an improvement in attitudes towards the provision of care for patients with alcohol use disorders in comparison to four out of the seven GPs in the control group. For the intervention group attitudes regarding role security and therapeutic commitment increased by 2.9 and 9.7 % respectively, whereas there was an increase in scores relating to attitudes on role security and therapeutic commitment of 4.6 and 1.75 % respectively for the control group.

**Table 5:** SAAPPQ measuring general practitioners’ attitudes towards the provision of care with those with alcohol use disorders and GPs’ perceived barriers to alcohol screening at baseline and follow up

	Intervention (*n* = 6)	Control (*n* = 7)
	Mean (SD)	Mean (SD)
Doctors' Attitudes Total SAAPPQ^a^ (SD)		
Baseline	50.7 (6.7)	54.4 (7.6)
Follow up	54 (6.8)	56 (6.3)
Doctors' Attitudes SAAPPQ: Role Security^b^ (SD)		
Baseline	23.2 (2.2)	21.9 (4.7)
Follow up	23.8 (3.8)	22.9 (1.2)
Doctors’ Attitudes SAAPPQ: Therapeutic Commitment^c^ (SD)		
Baseline	27.5 (5.6)	32.6 (4.3)
Follow up	30.2 (4.5)	33.1 (3.9)
Barriers Total Mean^d^ (SD)		
Baseline	15.2 (5.2)	16.2 (2)
Follow up	13.8 (1.6)	15.6 (1.6)
Lack of training in addiction		
Baseline	3.2 (1.5)	4 (1.1)
Follow up	3.6 (1.3)	4.3 (0.8)
Lack of time		
Baseline	2.8 (1.3)	2.3 (1.8)
Follow up	2.3 (1.8)	2.6 (0.5)
Lack of specialist staff		
Baseline	2.8 (1.8)	3 (1.4)
Follow up	1.8 (0.8)	2.9 (1.5)
Poor service availability		
Baseline	2.8 (1.7)	3.1 (1.1)
Follow up	2.3 (1.3)	2.7 (1.3)
Attitude of patient		
Baseline	3.5 (1.4)	3.3 (1.5)
Follow up	3 (1.5)	3.1 (1.2)

^a^Doctors’ Attitudes Total SAAPPQ - Short Alcohol and Alcohol Problems Perception Questionnaire’ (SAAPPQ). This 10-item, 7-point Likert-type questionnaire measured the attitudes of professionals towards the provision of care for patients with alcohol use disorders. Total scores range from 10 to 70, with lower scores indicative of more negative attitudes (refer to Additional file 1 for scoring code)

^b^Doctors' Attitudes SAAPPQ: Role Security - The role security domain within the SAAPPQ includes 2 sub-domains: role adequacy, and role legitimacy

^c^Doctors’ Attitudes SAAPPQ: Therapeutic Commitment - Therapeutic commitment involves motivation, task specific self-esteem, and work satisfaction

^d^Barriers Total Mean - GPs rated the importance of each of five barriers to alcohol screening, specifically: lack of training and education, lack of time, lack of specialist staff, poor service availability and attitude of patients. They rated their answers on a Likert scale from one to five (1 = most important and 5 = least important; composite score 5–25)

The composite score for perceived barriers to implementing SBI in general practice decreased for both groups from baseline to follow up with a greater decrease in the intervention group (–1.4 versus –0.6), with ‘lack of specialist support staff’ the barrier which was most affected (–1.0 versus –0.1).

When asked about which elements of the intervention they found useful, four (66.7 %) indicated they found hand-outs, guidelines, and remuneration ‘useful’ or ‘very useful’. These were followed by training (3, 50.0 %) and practice visits (2, 33.4 %). Overall, the mean composite score for all five intervention elements at participating practices was 10 (SD = 3.4, where 5 = very useful and 25 = not at all useful).

### Process / outcome measures

With respect to process measures, the proportion of patients who had (in the past three months) been screened for problem alcohol use was higher (53 % versus 26 %) in the intervention compared to control group (Table 6). This was also the case for the proportions who had received a brief alcohol intervention (47 % versus 19 %) and the proportion that had been referred for specialist alcohol treatment (3 % versus 0 %). In the intervention group, 22 people had an abnormal AUDIT-C at baseline, while 18 had an abnormal AUDIT-C at follow up (18 % reduction). In the control group, the number also fell from baseline to follow up (29 to 27, 7 % reduction). In the intervention group, while 14 people had an abnormal AUDIT at baseline, this fell to 8 at follow up (43 % reduction). In the control group, the number also fell from baseline to follow up (18 to 10, 44 % reduction). The intracluster correlation coefficient (ICC) and standard error for the proportion of patients with positive AUDIT-C results were 0.11 (SE = 0.013). The ICC will be used to determine sample size for the future definitive trial.

**Table 6:** Process / Outcome measures at follow up (and baseline where comparable) according to patient interviews

Process / outcome measure	Intervention (*n* = 34)	Control (*n* = 47)
	n (%)	n (%)
Alcohol screening * Q: Have you been asked about your alcohol use by healthcare professionals in the last 3 months?*	18 (52.9 %)	12 (25.5 %)
Brief Intervention * Q: Did they advise you on safe drinking or talk to you about alcohol in the last 3 months?*	16 (47.1 %)	9 (19.2 %)
Specialist referral * Q: Have you been referred to a specialist/ addiction counsellor for alcohol use in the last 3 months?*	1 (2.9 %)	0 (0 %)
Abnormal AUDIT-C		
At baseline	22 (66.7 %)	29 (61.7 %)
At follow up^a^	18 (54.5 %)	27 (57.5 %)
Abnormal AUDIT		
At baseline	14 (41.2 %)	18 (38.3 %)
At follow up	8 (23.5 %)	10 (21.3 %)

^a^ICC (SE) = 0.11 (0.013)

## Discussion

The study aimed to examine the feasibility and acceptability a complex intervention to enhance identification and treatment of problem alcohol use among people receiving opioid agonist treatment in primary care. Though not adequately powered to examine effectiveness the follow-up alcohol screening rates in the intervention group were higher than in the control group (53 % versus 26 %), as were alcohol brief intervention rates (47 % versus 19 %). The follow-up prevalence of problem alcohol use in the intervention group (measured by AUDIT-C) dropped by 18 %, compared to seven per cent in the control group.

The key parameters identified from this study for a definitive cluster randomised controlled trial (RCT) to determine the effectiveness of this intervention were GP recruitment rate (13 %), patient recruitment rate (81 %), GP retention rate (81 %) and patient retention (63 %).

The GP recruitment rate was lower than planned. Recruitment issues are echoed in other primary care research [28, 29]. Recruitment in primary care has particular challenges related to the characteristics of primary care practitioners, their patients and the dispersed nature of clinics [30]. Additional challenges, such as physical, psychological co-morbidity and adverse social circumstances [31] in conjunction with practices located in deprived areas, should be considered when planning RCTs involving this group of patients.

Our GP recruitment strategy targeting socio-economically deprived areas in the Dublin Mid-Leinster and the Mid-West regions and use of postal invitations, limited our recruitment rate and undermined the generalizability of our findings. Although, there has been significant growth in prevalence of opiate users outside of Dublin [32], the lower number of patients receiving opioid agonist treatment in the Mid-West region resulted in a disproportionately lower acceptance rate than the Dublin-Mid Leinster region. Future strategies will encompass a larger sample size targeting a greater proportion of MMT (methadone maintenance treatment) GPs in Ireland and the implementation of a more rigorous recruitment process. We will (i) Engage with networks of practices (thematic or geographical) with an interest in conducting research and development in MESUDs (Mental Health Substance Use Disorders) as a key priority; (ii) Develop a relationship with practice staff and ensure adequate practice support to enhance recruitment and retention of practitioners and patients; and (iii) Implement active recruitment measures, involving personal contact between researchers and practitioners that avoids mailing unsolicited information, which may also encourage participation.

Following a review of qualitative interviews with GPs, time constraints were identified as the primary obstacle in the implementation of the intervention. In addition, some GPs tended to overestimate their competency in detecting problem alcohol use relying on visual and verbal cues from patients rather than screening patients routinely. This highlights the need for greater researcher support for GPs, improved study format and delivery of educational intervention to improve SBI rates among this cohort of patients.

Positive attitudes towards caring for patients with problem alcohol use were observed among participating GPs, with the SAAPPQ score improving in both intervention and control groups at follow up, and to a greater extent in the intervention group, particularly in relation to therapeutic commitment. Though the improved SAAPPQ score was modest, this is nonetheless important as attitudes are important predictors of GPs’ involvement in managing alcohol problems (especially in role security and therapeutic commitment domains) [33]. The CME-approved educational workshops to enable GPs to screen for or treat problem alcohol use among people receiving opioid agonist treatment were also received favourably. Most useful components of the training were hand-outs, guidelines and remuneration. The findings suggest GPs found the intervention useful. This is consistent with other research that indicates training GPs in the management of alcohol use disorders among people receiving opioid agonist treatment is likely to be of use to GPs [18] and perhaps patients.

The assessment of perceived barriers to alcohol screening suggested that training heightened GPs’ awareness of how to manage problem alcohol use and focused their attention on how to address the practicalities of implementing it in their everyday consultations. The need for specialist staff was the most important barrier further highlighting the importance of implementing the provision of additional support and resources for a less motivated cohort of GPs.

Qualitative interviews found that while all GPs found the intervention informative and feasible, most considered it challenging to incorporate into practice. Barriers included time constraints, and overlooking and underestimating problem alcohol use among this cohort of patients. Patients reported that (in the absence of the intervention) their use of alcohol was rarely discussed with their GP, and were reticent to initiate conversations on their alcohol use for fear of having their methadone dose reduced. The findings suggested that while a complex intervention seems feasible and acceptable, the barriers highlighted must be overcome to enable consistent, regular, and accurate screening by GPs.

Although screening for problem alcohol use is a prevention priority in primary care for adults (especially high risk groups such as people receiving opioid agonist treatment [21]), thus far it has one of the lowest delivery rates, with screening or intervention typically completed only when a risk factor is evident [34]. Our study suggests that if adequately supported, primary care providers can screen and intervene more, based on our biased, small sample size. Furthermore education of GPs on the importance of screening this cohort of patients and the integration of the screening instrument within electronic medical records can improve treatment for at risk groups [35, 36].

The decrease of drinking in the control group is consistent with improvements that have been observed in intervention trials targeting various health behaviours and may occur in response to undergoing baseline assessment, participants’ awareness of being involved in experimental studies, or due to the delivery of a more intensive ‘usual care’ than would be typically experienced [37, 38]. Given the interaction with practices in the ‘control’ group, this is most likely what improved outcome measures in this group.

This feasibility study was not designed to determine effectiveness and thus inferences on the likely efficacy of the intervention should be interpreted with caution. Furthermore, it is likely that we recruited practices and patients who were more positively disposed toward the intervention. While it is evident that the final 15 GPs out of an original pool of 149 represented a biased, self-selected sample, the findings are still of value because we followed guidelines on feasibility studies and we achieved our objectives, i.e. estimating sample size for a future definitive trial, testing integrity of study protocol, data collection instruments, randomisation procedure, recruitment, consent, acceptability of intervention and identification of most appropriate primary outcome measure.

It appeared that the poor rate of GP participation was directly connected to the complexity of the study protocol, the intervention, the multiple data collection and measurement instruments. The GP recruitment method and the proposed intervention did not appear to be sufficiently attractive in the eyes of the busy practitioners, despite the financial incentive offered to them. The recognition of the importance of the intervention was sufficient to attract participating practitioners to the dedicated CME sessions; nonetheless, for a definitive study, the research protocol and instruments should be simplified and modified / minimised to increase acceptability to GPs and to improve GP recruitment rate.

## Conclusion

A complex intervention to support alcohol screening and brief intervention among people receiving opioid agonist treatment in primary care appears feasible. Future research should address improving the complex intervention to provide additional support to GPs in making SBIRT a consistent part of their treatment for people receiving opioid agonist treatment. Strategies that maximise and enhance recruitment (e.g. focussed engagement with practices, targeting deprived regions, etc.) will be integral to this study protocol. Enhanced contact and interaction of researchers with GPs during the recruitment process may encourage more active involvement of GPs.

## Additional file

Additional file 1: Short Alcohol and Alcohol Problems Perception Questionnaire. Questions designed to explore the attitudes of staff working with people with alcohol use disorders. (DOC 47 kb)

## Abbreviations

AUDIT: Alcohol use disorders identification test
AUDIT-C: Alcohol use disorders identification test – consumption
BI: Brief intervention
CME: Continuing medical education
FRAMES: Feedback, responsibility, menu of strategies, empathy, self-efficacy
GP: General practitioner
HSE: Health service executive
ICC: Intracluster correlation coefficient
ICGP: Irish College of General Practitioners
MESUDs: Mental health substance use disorders
MMT: Methadone maintenance treatment
MRC: Medical research council
PINTA: *P*sychosocial *INT*erventions for problem *A*lcohol use
RCT: Randomised controlled trial
SAAPPQ: Short alcohol and alcohol problems perception questionnaire
SBI: Screening and brief intervention
SBIRT: Screening and brief intervention and referral to treatment
SE: Standard error
SSPS: Statistical packages for the social science
WHO: World Health Organisation.
