# Supplementary material for: Feasibility of alcohol screening among patients receiving opioid treatment in primary care
Source: BMC Fam Pract. 2016 Nov 5;17:153. doi: 10.1186/s12875-016-0548-2 (PMC5097838; doi:10.1186/s12875-016-0548-2)
Supplement: Additional file 1: — Short Alcohol and Alcohol Problems Perception Questionnaire. Questions designed to explore the attitudes of staff working with people with alcohol use disorders. (DOC 47 kb) [file 12875_2016_548_MOESM1_ESM.doc]

**Short Alcohol and Alcohol Problems Perception Questionnaire**

The questions in this section are designed to explore the attitudes of staff working with people with alcohol use disorders. There are no right or wrong answers. Please indicate the extent to which you agree or disagree with the following statements:

1 = Strongly agree

2 = Quite strongly agree

3 = Agree

4 = Neither agree or disagree

5 = Disagree

6 = Quite strongly disagree

7 = Strongly disagree

|  |  | **Strongly agree** | **Quite strongly agree** | **Agree** | **Neither agree or disagree** | **Disagree** | **Quite strongly disagree** | **Strongly disagree** |
| --- | --- | --- | --- | --- | --- | --- | --- | --- |
|  |  | **1** | **2** | **3** | **4** | **5** | **6** | **7** |
| 1 | I feel I know enough about causes of drinking problems to carry out my role when working with drinkers |  |  |  |  |  |  |  |
| 2 | I feel I can appropriately advise my patients about drinking and its effects |  |  |  |  |  |  |  |
| 3 | I feel I do not have much to be proud of when working with drinkers |  |  |  |  |  |  |  |
| 4 | All in all I am inclined to feel I am a failure with drinkers |  |  |  |  |  |  |  |
| 5 | I want to work with drinkers |  |  |  |  |  |  |  |
| 6 | Pessimism is the most realistic attitude to take towards drinkers |  |  |  |  |  |  |  |
| 7 | I feel I have the right to ask patients questions about their drinking when necessary |  |  |  |  |  |  |  |
| 8 | I feel that my patients believe I have the right to ask them questions about drinking when necessary |  |  |  |  |  |  |  |
| 9 | In general, it is rewarding to work with drinkers |  |  |  |  |  |  |  |
| 10 | In general I like drinkers |  |  |  |  |  |  |  |

**Thank you for taking the time to complete this survey**

**Scoring**

*Reverse scoring for items 1, 2, 5, 7, 8, 9, 10*

*Role adequacy: Add scores on items 1, 2*

*Role legitimacy: Add scores on items 7, 8*

*Motivation: Add scores on items 5, 6*

*Task-specific self-esteem: Add scores on items 3, 4*

*Work satisfaction: Add scores on items 9, 10*

*Role security: Add scores on role adequacy and role legitimacy*

*Therapeutic commitment: Add scores on Motivation, Work Satisfaction and Task-specific Self-esteem*
